# The role of mRNA m^6^A methylation in the nervous system

**DOI:** 10.1186/s13578-019-0330-y

**Published:** 2019-08-20

**Authors:** Jiashuo Li, Xinxin Yang, Zhipeng Qi, Yanqi Sang, Yanan Liu, Bin Xu, Wei Liu, Zhaofa Xu, Yu Deng

**Affiliations:** School of Public Health, China Medical University, Shenyang, 110122 Liaoning China

**Keywords:** Epitranscriptomics, N6-methyladenosine (m^6^A), Methyltransferase, Demethylase, Binding protein, Nervous system

## Abstract

Epitranscriptomics, also known as “RNA epigenetics”, is a chemical modification for RNA regulation. Ribonucleic acid (RNA) methylation is considered to be a major discovery following the deoxyribonucleic acid (DNA) and histone methylation. Messenger RNA (mRNA) methylation modification accounts for more than 60% of all RNA modifications and N6-methyladenosine (m^6^A) is known as one of the most common type of eukaryotic mRNA methylation modifications in current. The m^6^A modification is a dynamic reversible modification, which can directly or indirectly affect biological processes, such as RNA degradation, translation and splicing, and can play important biological roles in vivo. This article introduces the mRNA m^6^A methylation modification enzymes and binding proteins, and reviews the research progress and related mechanisms of the role of mRNA m^6^A methylation in the nervous system from the aspects of neural stem cells, learning and memory, brain development, axon growth and glioblastoma.

## Background

Epitranscriptomics, also known as “RNA epigenetics”, is a chemical modification for RNA regulation [1]. According to its function, RNA can be divided into two broad categories, including encoding protein mRNA and non-coding RNA. With the deep research of epitranscriptomics, the researchers found methylation modification on mRNA, which is involved in the regulation of eukaryotic gene expression [2–4].

**Fig. 1:**
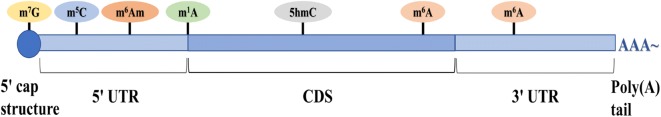
Different types of mRNA methylation modification

The mRNA is a type of RNA with genetic information synthesized by DNA transcription, which acts as a template in protein synthesis and determines the amino acid sequence of the peptide chain [5]. It is an important RNA in the human body. The methylation is the process of catalytically transferring a methyl group from an active methyl compound such as S-adenosylmethionine (SAM) to another compound, which can chemically modify certain proteins or nucleic acids to form a methylated product [6]. In biological systems, methylation influences heavy metal modification, regulation of gene expression, regulation of protein function, RNA processing, etc. [7]. At the early 1970s, scientists discovered the presence of the methylation modification in mRNA [8, 9]. The mRNA methylation modification mainly located in the nitrogen atom of the base group to form m^6^A, which is enriched in long exons and overrepresented in transcripts with alternative splicing variants [10]. The mRNA methylation modifications also include 5-methylcytosine (m^5^C), N1-methyladenosine (m^1^A), 5-hydroxymethylcytosine (5hmC), N6, 2′-*O*-dimethyladenosine (m^6^Am), 7-methylguanine (m^7^G) (Fig. 1). These modifications can affect regulation of various biological processes, such as RNA stability and mRNA translation, and abnormal mRNA methylation is linked to many diseases [11].

## Main text

### Discovery and distribution of m^6^A

The m^6^A is the most common and abundant methylation modification in mRNA [12, 13]. In 1974, Desrosie used the polyadenosinic acid (PolyA) structure in eukaryotes, to discover the methylation status of mRNA in hepatoma cells, and found that the main methylation modification in mRNA was m^6^A (approximately 80%) [8]. In addition, the presence of m^6^A was also detected in a variety of eukaryotes and viral mRNA [14].

**Fig. 2:**
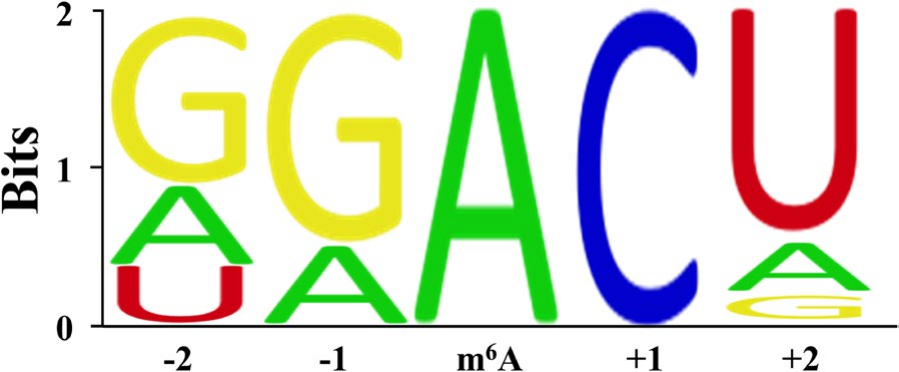
RRACH sequence

In mammals, m^6^A is widely distributed in multiple tissues. Studies by Meyer showed that m^6^A expression was higher in liver, kidney and brain than in other tissues. It has also been found that the content of m^6^A is very different in various cancer cell lines [15]. With the help of high-throughput sequencing technology, a rough m^6^A modification map has been obtained. Meyer studied the m^6^A modification in mouse brain and found that it was mainly distributed inside the gene (94.8%), where the proportions in the protein coding region (CDS), untranslated regions (UTRs) and introns are 50.9%, 41.9%, and 2.0% respectively [16]. The m^6^A in the UTRs region tends to be enriched in the 3′UTR, while in the CDS region it is mainly enriched near the stop codon [17]. The m^6^A modification occurs mainly on the adenine in the RRACH sequence, where R is guanine or adenine, and H is uracil, adenine or cytosine [18] (Fig. 2).

### mRNA m^6^A methylation modification enzyme

The methylation modification of m^6^A has been proved to be reversible, as it involves both methyltransferase and demethylase. The main role of methyltransferases is to catalyze the m^6^A modification of mRNA, while demethylases act on demethylation of bases that have had m^6^A modification [19, 20].

#### m^6^A methyltransferase

The m^6^A methyltransferase, also known as “Writers”, is an important kind of catalytic enzymes [21]. Methyltransferase like 3/14 (METTL3/14), Wilms’ tumour 1-associating protein (WTAP), KIAA1429 and RNA binding motifs protein 15/15B (RBM15/15B) are core components of the m^6^A methyltransferase, which form complexes that work together to perform catalytic functions. Besides, E3 ubiquitin-protein ligase Hakai (HAKAI) and zinc finger CCCH-type containing 13 (ZC3H13) are also the part of the mRNA methyltransferase complex.

The METTL3 is identified as a SAM-binding component of the complex and has its own catalytic ability, which is highly conserved in eukaryotes [22]. METTL14 is closely homologous to METTL3. It does not bind to the SAM domain and does not with independently m^6^A methyltransferase function. Biochemical characterization has shown that METTL3 and METTL14 proteins form a stable complex with a stoichiometric ratio of 1:1, and the methylation activity of the complex is higher than that of METTL3 alone. Among them, METTL3 is a catalytically active subunit, and METTL14 plays a key role in substrate identification [23, 24].

The WTAP is a regulatory subunit of the m^6^A methyltransferase complex, which can interact with METTL3 and METTL14. Knocking out WTAP can significantly reduce the m^6^A peak in cellular mRNA, even more effective than knocking down METTL3 or METTL14. The WTAP-bound gene has a change in alternative splicing patterns [25].

The KIAA1429, also known as vir-like m^6^A methyltransferase associated (VIRMA), is a homologous protein of the Virilizer protein in Drosophila, which is closely related to the methyltransferase complex. The N-terminus of KIAA1429 has the ability to gather methyltransferase-catalyzed core METTL3/METTL14/WTAP that can achieve the regulation of fixed-point m^6^A levels on mRNA [26].

It was identified by co-immunoprecipitation that the binding of RBM15/15B at the RRACH sequence site is three to fourfold o higher than that at the non-methylation site. Knocking down the RBM15 or RBM15B alone can reduce the m^6^A levels in cellular mRNA, and knocking down both RBM15 and RBM15B can result in a significant decrease of the m^6^A levels in mRNA [27].

The HAKAI, also known as CBL proto-oncogene like 1(CBLL1), is an E3 ubiquitin ligase. Down-regulation of the HAKAI in Arabidopsis can result in a decrease in m^6^A level [28]. ZC3H13 is also an important component of the methyltransferase complex and is key to anchor the complex in the nucleus [29]. Methyltransferase like 16 (METTL16) is a m^6^A methyltransferase of the mRNA precursor that maintains SAM homeostasis by regulating alternative splicing of methionine adenosyltransferase II alpha (MAT2a) [30–32].

#### m^6^A demethylase

The m^6^A demethylase, also known as the “Erasers”. In eukaryotes, m^6^A demethylases are fat mass and obesity-associated protein (FTO) and alkB homolog 5 (alkB homolog 5, ALKHB5). In Arabidopsis, the alkB homolog 10B (ALKHB10B) has also been found as a m^6^A demethylase of mRNA.

The FTO also known as alkB homolog 9 (ALKBH9), which is a member of the Alkb protein family and associated with obesity. FTO is the first-discovered RNA demethylase. The long stem loop domain at the C-terminus of FTO enables the FTO proteins demethylate [33, 34].

The ALKBH5 is another protein of the AlkB family and plays an important regulatory role in biological processes, such as mRNA processing. The ALKBH5 is similar to FTO and is also a Fe^2+^ and α-Ketoglutaric acid dependent non-heme oxygenase. The ALKBH5 has an alanine-rich region at the N-terminus and a unique coiled-coil structure. It only demethylates the m^6^A modification on single-stranded RNA/DNA, and the catalytic reaction removes methyl groups directly from m^6^A-methylated adenosine instead of oxidative demethylation [35, 36].

The ALKBH10B is an m^6^A demethylase of mRNA in Arabidopsis, which regulates mRNA stability and affects the transformation of Arabidopsis from vegetative growth to reproductive growth [37].

### mRNA m^6^A methylation binding protein

The m^6^A-modified mRNA that performs a specific biological function requires a specific RNA-binding protein-readers. Binding assays of RNA protein in vitro have identified a variety of binding proteins, including YTH domain containing RNA binding protein (YTP), heterogeneous nuclear ribonucleoprotein (hnRNP), eukaryotic initiation factor 3 (eIF3), Insulin-like growth factor 2 mRNA-binding protein (IGF2BP) and Proline rich coiled-coil 2A (Prrc2a). The functions of these binding proteins mainly include specific binding to the m^6^A methylation region, weakening the homologous binding to RNA reading proteins, and altering the secondary structure of RNA to alter protein–RNA interaction [38, 39].

YTH domain containing RNA binding protein include YTH domain-containing family protein 1/2/3 (YTHDF1/2/3) and YTH domain-containing protein 1/2 (YTHDC1/2). YTHDF1/2/3 and YTHDC2 specifically recognize the m^6^A-modified mRNA in the cytoplasm, while the recognizing sites of YTHDC1 are mainly in the nucleus. These proteins all have a YTH domain at the C-terminus. They are capable of overlapping with the m^6^A RRACH fragment to mediate RNA-specific binding, while its proline/glutamine/asparagine enrichment (P/Q/N-rich) domain is related to subcellular localization [40, 41].

YTHDF1 is combined with translation initiation factors and ribosomes, improving the translation efficiency. YTHDF2 is the first-discovered binding protein. Specifically, it recognizes and binds m^6^A-containing RNAs, and regulates mRNA stability [42, 43]. YTHDF3 promotes the translation of mRNA and regulates the mRNA stability. YTHDF3 and YTHDF1 coordinately control during translation [44, 45]. YTHDC1 regulates the mRNA cleavage by recruiting splicing factors [46–48]. YTHDC2 accelerates the degradation of the modified mRNA and enhances the translation of the corresponding protein by recognizing m^6^A [49].

The hnRNP is a group of RNA-binding proteins that contain nearly 30 nucleic acid-binding proteins with molecular weights ranging from 30 to 120 kDa, which can interact with each other to form the complex, where A1, A2, B1, B2, C1 and C2 are the main core components. Heterogeneous nuclear ribonucleoprotein A2/B1 (HNRNPA2B1) is capable of specifically recognizing the m^6^A modifications on transcripts, activating downstream variable shear events of partial genes [50, 51]. Heterogeneous nuclear ribonucleoprotein C (HNRNPC) is responsible for recognizing the m^6^A modifying group and mediating the processing of the mRNA precursor in the nucleus, affecting the abundance and alternative splicing of target transcripts. The m^6^A can increase the accessibility of its surrounding RNA sequences binding to heterogeneous nuclear ribonucleoprotein G (HNRNPG). In the transcriptome, the m^6^A site regulates RNA-HNRNPG interactions to alter target mRNA expression and alternative splicing patterns [52].

**Fig. 3:**
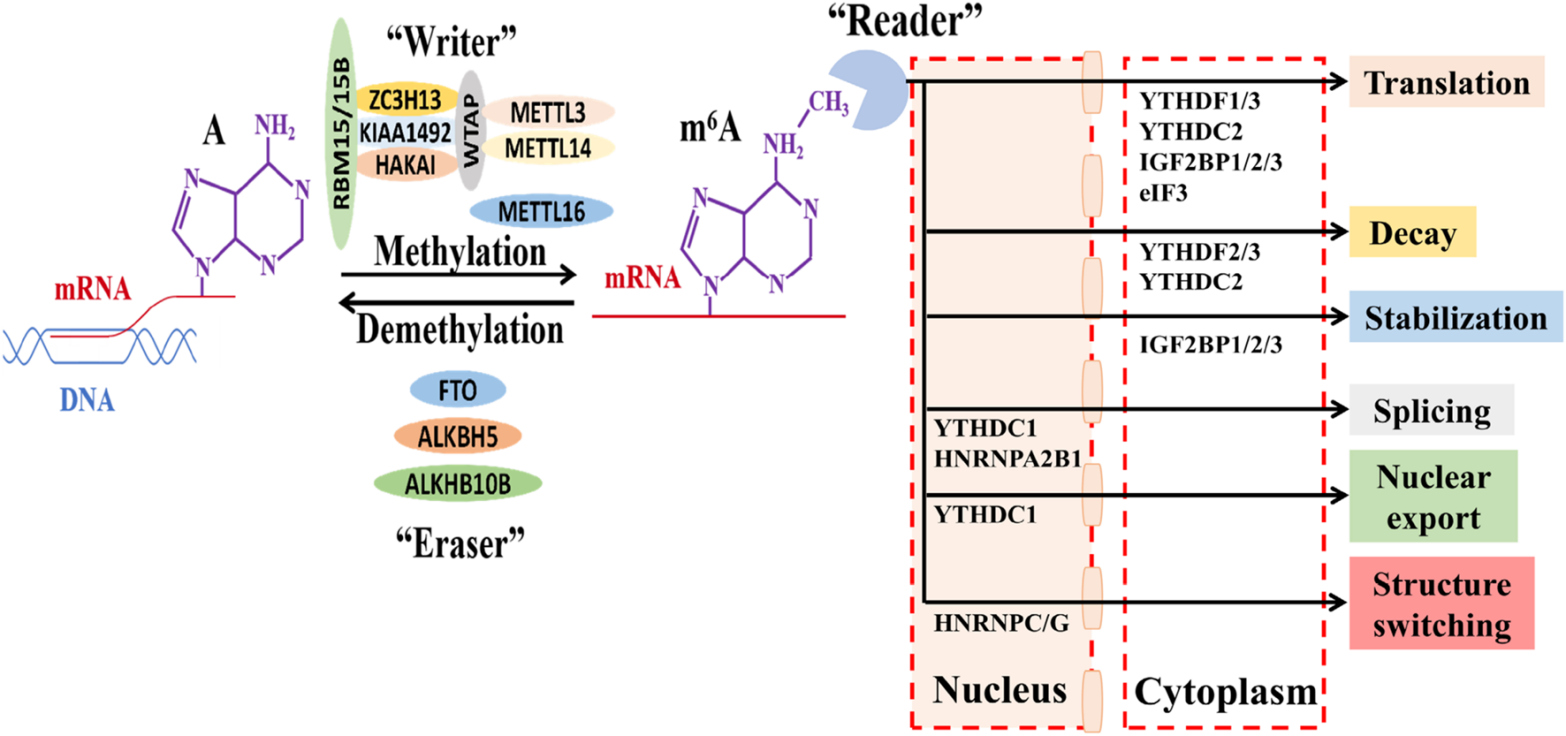
mRNA m^6^A methylation-associated protein

In mammalian cells, eIF3 is the largest eukaryotic initiation factor and plays a key role in the initiation of eukaryotic translation. It is able to directly bind to the 5′UTR m^6^A of mRNA, thereby facilitating translation of mRNA. IGF2BP protein is a unique m^6^A reader. The family mainly includes IGF2BP1/2/3. IGF2BP can make the target gene and corresponding translation more stable [53]. Prrc2a is a new m^6^A reader that stabilizes mRNA expression by binding to a consensus GGACU motif in the CDS region in an m^6^A-dependent manner [54] (Fig. 3).

### Neurobiological function of mRNA m^6^A methylation

Sequencing results have showed that the level of RNA m^6^A modification increase significantly during embryogenesis [55–58]. Compared to other organs or tissues, the overall level of m^6^A in the head is significantly higher. This suggests that the mRNA m^6^A modification has potential neurobiological functions in the nervous system and is worthy of further study [59–63].

#### Effect of m^6^A on neural stem cells

Neural stem cells maintain cell populations through self-renewal, and can differentiate into various nerve cells such as neurons, astrocytes, and oligodendrocytes [64–67]. A number of studies have shown that the mRNA m^6^A modification can affect the self-renewal and differentiation of neural stem cells [68–70]. These new findings will promote stem cell therapy and gene-targeted therapy for neurological diseases.

Inactivation of Mettl3 in mouse and human embryonic stem cells leads to a decrease in m^6^A, and severely impairs the transition of neurons from self-renewal to differentiation. The knockout of Mettl3 can cause early embryonic lethality and impair the formation of mature neurons in the embryoid body [58, 71]. Wang et al. found that when knocking out Mettl14 in embryonic neural stem cells in a mouse model, the proliferation of neural stem cells was significantly reduced and differentiated prematurely. It indicates that m^6^A can promote the proliferation of neural stem cells and prevent premature differentiation of cells, thus ensuring the reserve of neural stem cell bank [72, 73]. Mettl14 and Mettl3 can participate in neurogenesis by regulating the cell cycle progression of cortical neural stem cells, which acts in a m^6^A-dependent way [74]. The SMAD2/3 protein binds to the METTL3-METTL14-WTAP complex and promotes the differentiation of embryonic stem cells into neuroendodermal cells [75].

The Ythdf2-mediated m^6^A mRNA clearance has a regulatory effect on neurodevelopment in mice. Proliferation and differentiation of neural stem cells are seriously affected by the deletion of embryonic Ythdf2 [76].

#### Effect of m^6^A on learning and memory

In the emerging field of epitranscriptomic mechanisms, mRNA m^6^A modification has potential role in learning and memory [77]. It regulates physiological and stress-induced behavior in the adult mammalian brain, and augments the strength of weak memories [78–80]. As a newly identified element in the region-specific gene regulatory network in the mouse brain, mRNA m^6^A modification plays a vital role in the death of dopaminergic neuron [81, 82].

Mettl3-mediated RNA m^6^A modification has the direct effect on regulating hippocampal-dependent long-term memory formation. The decrease of Mettl3 in the mice hippocampus may reduce its memory consolidation, and adequate training or restoration would restore the ability of learn and memory. The abundance of Mettl3 in the hippocampus of wild-type mice is positively correlated with learning efficiency, and the overexpression of Mettl3 can significantly enhance the long-term memory consolidation [83]. METTL14 is critical for striatum function and transcriptional regulation of learning epitopes. In cell experiments, the deletion of METTL14 reduces striatum m^6^A levels without altering cell number or morphology, increases neuronal excitability and severely impaired striatal-mediated behavior [84].

Fto can regulate the activity of dopaminergic midbrain circuits. Inactivation of the Fto gene weaken neuronal activity and behavioral responses that are dependent on dopamine receptor type 2 (D2R) and type 3 (D3R) (collectively D2-like receptors) [85]. FTO also regulates dopaminergic neurotransmission deficits caused by arsenite [86]. Walters [87] has found that Fto plays an important role in the formation of mouse hippocampal-dependent memory. The decrease in Fto protein observed shortly after the situational fear reflex indicates that Fto typically limits memory formation. The m^6^A is regulated in the activity-dependent way in the adult brain, and may fine-tune mRNA turnover during memory-related processes [88]. When knocking out the Fto gene in the prefrontal cortex of mice, the intensity of m^6^A on several fear-related genes in neurons increases significantly, and the knockdown of Fto further enhances the consolidation of fear memory [89]. FTO plays important roles in learning and memory. The loss of FTO led to the altered expression of several key components of the brain derived neurotrophic factor pathway that were marked by m^6^A [90].

In the adult mouse hippocampus, the m^6^A binding protein Ythdf1 can promote neuronal stimulation of protein translation of target transcripts, thereby facilitating learning and memory. Mice with a genetic deletion of Ythdf1 have showed the deficits of learning and memory, impaired hippocampal synaptic transmission and long-term potentiation [91]. Prrc2a controls the specification and myelination of oligodendrocyte, and Prrc2a knockout induces cognitive defects in a mouse model [54].

#### Effect of m^6^A on brain development

Widespread and dynamic m^6^A methylation were identified in the developing mouse cerebellum. RNA m^6^A methylation is controlled in a precise spatiotemporal manner and participates in the regulation of postnatal development of the mouse cerebellum [92, 93].

Specific inactivation of Mettl3 in mouse nervous system causes severe developmental defects in the brain. Mettl3-mediated m^6^A participates in cerebellar development by controlling mRNA stability of genes involved in cerebellar development and apoptosis [94].

Under the low pressure and hypoxia, the level of RNA m^6^A methylation in the cerebellar of Alkbh5-deficient mouse is imbalanced, which leads to an increase in the efficiency of extranuclear RNA excretion and a significant change in cerebellar phenotype, including neuronal structural disorder, abnormal cell proliferation and differentiation, and other phenotypes [92].

#### Effect of m^6^A on synaptic growth

The m^6^A modification plays a key role in synaptic regeneration of mature mouse neurons. Increased m^6^A in somatic neurons alters the transcriptome response to synaptic plasticity [77, 89]. The m^6^A methylation of neurological function-related genes in the hippocampus of human immunodeficiency virus transgenic rats is significantly different, suggesting synaptic damage and neurodegeneration [95]. The m^6^A methylation of synaptic mRNAs critically contribute to synaptic function in healthy adult mouse forebrains [96].

Deletion of Mettl14 reduces functional axonal regeneration in the peripheral nervous system of the body. After knockdown of Mettl14, the axonal regeneration of retinal ganglion neurons in the central nervous system is also diminished [97].

The m^6^A modification can affect axon growth by regulating local translation of mRNA in neuronal axons. FTO is highly expressed in axons of neurons. Local translation in axons plays an important role in neurodevelopment, including axon guidance, axon growth, and neuronal specifications [90, 98].

The mRNA m^6^A modification of synaptic plays a key role in synaptic function. After knocking out the dendritic positioning readers Ythdf1 and Ythdf3 in cultured hippocampal neurons, m^6^A-reader-deficient neurons have abnormal spine morphology and the spines are reduced. Knocking out the Ythdf1 gene of mouse, in the peripheral and peripheral nervous system, functional axon regeneration is reduced [97, 99]. The neurons of YTHDF2^−/−^ could not produce normal synapses [76].

#### Effect of m^6^A on glioblastoma

Several studies have revealed the role of m^6^A witers and erasers in glioblastoma. Changes of the m^6^A level in glioblastoma stem cell-like cells (GSC) severely affect the growth, self-renewal and development of tumor. The mRNA m^6^A methylation is expected to be a new target for the treatment of glioblastoma [100].

Decreasing the m^6^A levels by knocking down METTL3 and/or METTL14 enhance growth and self-renewal of GSCs in vitro, and promote the ability of GSCs to form brain tumors in vivo. The Mettl3-mediated m^6^A modification plays a key role in neurosphere maintenance and glioma cell dedifferentiation [101–103]. Ethyl form of methylbenzoic acid (MA2) is a selective inhibitor of FTO, which can significantly inhibit tumor progression and prolong the lifespan of GSC mice. Therefore, The Fto may play a key carcinogenic role in GSC self-renewal and is required for the development of glioblastoma [101]. ALKBH5 is able to maintain stem cell in malignant glioma cells, and ALKBH5-mediated m^6^A modification on forkhead box M1 (FOXM1) mRNA is involved in the maintenance of tumor stem cell. High expression of ALKBH5 predicts poor prognosis in glioblastoma patients [104, 105] (Table 1).

**Table 1:** Neurobiological functions of mRNA m^6^A methylation

Neurological disease	Related enzymes and proteins
Neural stem cell	METTL3, METTL14 and YTHDF2
Learning memory	METTL3, METTL14, FTO, YTHDF1 and Prrc2a
Brain development	METTL3 and ALKBH5
Synaptic growth	METTL14, FTO, YTHDF1, YTHDF2 and YTHDF3
Glioblastoma	METTL3, METTL14, FTO and ALKBH5

## Conclusion

In summary, the mRNA methylation is an important epitranscriptomic modification and the m^6^A is highly expressed in the brain. The mRNA m^6^A methylation has a wide range of effects on the nervous system, and plays an important part in self-renewal of neural stem cells, learning memory, brain development, synaptic growth and proliferation of glioma cells. This new regulatory system will promote targeted therapy for neurological diseases.

However, mRNA m^6^A methylation is a relatively new field and many problems remain unknown. Up till now, all of the demethylases found belong to the AlkB family, and whether other proteins in the AlkB family are also involved in mRNA demethylation is worthy for further study. HNRNPA1, HNRNPG and HNRNPM play a key role in the methylation of protein arginine. These proteins are similar to HNRNPA2B1 and HNRNPC, and belong to the hnRNP binding protein family. It is worth exploring its role in mRNA m^6^A methylation.

Variations in the FTO gene can not only regulate D2R-dependent reward learning [106–108], but also affect nerve adjust food visual, produce more frequent rewards [109–111], affect the control of mood and impulse [112–114], and affect obesity by regulating brain signaling pathways [115, 116]. The homozygous mutation of FTO gene can reduce the brain capacity of healthy elderly people, increase the susceptibility to brain atrophy during aging, and even affect the brain volume of adolescents [117, 118]. The genetic polymorphism of FTO is related to attention-deficit/hyperactivity disorder (ADHD), Alzheimer’s disease and depression [119–124]. Whether it is as demethylase that affects these diseases, is worthy of further study.

The genetic polymorphism of ZC3H13 is associated with schizophrenia. Nito, another member of the m^6^A methyltransferase complex in Drosophila, called RBM15 in human, controls the axonal growth and differentiation and regulates the synapse formation through neuronal activity. Whether human ZC3H13 and RBM15 genes have the effect on synaptic growth, is worthy of further study.

To study the methylation mechanism of mRNA m^6^A and find potential targets for treatment, it is hopeful to develop inhibitors or agonists of related proteins for clinical treatment in the future.

## Data Availability

Not applicable.

## Abbreviations

5hmC: 5-hydroxymethylcytosine
ADHD: attention-deficit/hyperactivity disorder
ALKHB5/9/10B: alkB homolog 5/9/10B
CBLL1: CBL proto-oncogene like 1
CDS: coding region
D2/3R: dopamine receptor type 2/3
DNA: deoxyribonucleic acid
eIF3: eukaryotic initiation factor 3
FOXM1: forkhead box M1
FTO: fat mass and obesity-associated protein
GSC: glioblastoma stem cell-like cells
HAKAI: E3 ubiquitin-protein ligase Hakai
hnRNP: heterogeneous nuclear ribonucleoprotein
HNRNPA2B1/C/G: heterogeneous nuclear ribonucleoprotein A2B1/C/G
IGF2BP: insulin-like growth factor 2 mRNA-binding protein
m^1^A: N1-methyladenosine
m^5^C: 5-methylcytosine
m^6^A: N6-methyladenosine
m^6^Am: N6, 2′-*O*-dimethyladenosine
m^7^G: 7-methylguanine
MA2: methylbenzoic acid
MAT2a: methionine adenosyltransferase II Alpha
METTL3/14/16: methyltransferase like 3/14/16
mRNA: messenger RNA
P/Q/N-rich: proline/glutamine/asparagine enrichment
PolyA: polyadenosinic acid
Prrc2a: proline rich coiled-coil 2A
RBM15/15B: RNA binding motifs protein 15/15B
RNA: ribonucleic acid
SAM: S-adenosylmethionine
UTRs: untranslated regions
VIRMA: vir-like m^6^A methyltransferase associated
WTAP: wilms’ tumour 1-associating protein
YTHDC1/2: YTH domain-containing protein 1/2
YTHDF1/2/3: YTH domain-containing family protein 1/2/3
YTP: YTH domain containing RNA binding protein
ZC3H13: zinc finger CCCH-type containing 13
